# Steady-State Analysis of Genetic Regulatory Networks Modelled by Probabilistic Boolean Networks

**DOI:** 10.1002/cfg.342

**Published:** 2003-12

**Authors:** Ilya Shmulevich, Ilya Gluhovsky, Ronaldo F. Hashimoto, Edward R. Dougherty, Wei Zhang

**Affiliations:** 1 Cancer Genomics Laboratory University of Texas M. D. Anderson Cancer Center 1515 Holcombe Blvd. Unit 85 Houston TX 77030 USA; 2 Sun Microsystems Laboratories Palo Alto CA USA; 3 Department of Electrical Engineering Texas A&M University College Station TX 77843 USA; 4 Departamento de Ciencia de Computacao Universidade de Sao Paulo Sao Paulo Brazil

## Abstract

Probabilistic Boolean networks (PBNs) have recently been introduced as a promising class of models of genetic regulatory networks. The dynamic behaviour of PBNs can be analysed in the context of Markov chains. A key goal is the determination of the steady-state (long-run) behaviour of a PBN by analysing the corresponding Markov chain. This allows one to compute the long-term influence of a gene on another gene or determine the long-term joint probabilistic behaviour of a few selected genes. Because matrix-based methods quickly become prohibitive for large sizes of networks, we propose the use of Monte Carlo methods. However, the rate of convergence to the stationary distribution becomes a central issue. We discuss several approaches for determining the number of iterations necessary to achieve convergence of the Markov chain corresponding to a PBN. Using a recently introduced method based on the theory of two-state Markov chains, we illustrate the approach on a sub-network designed from human glioma gene expression data and determine the joint steadystate probabilities for several groups of genes.


**Comparative and Functional Genomics**
*Comp Funct Genom* 2003; **4**: 601–608.
Published online in Wiley InterScience (www.interscience.wiley.com). **DOI:** 10.1002/cfg.342

**Research Article**

### Steady-state analysis of genetic regulatory networks modelled by probabilistic Boolean networks

Ilya Shmulevich[1]*, Ilya Gluhovsky[2], Ronaldo F. Hashimoto[4,3], Edward R. Dougherty[3,1] and Wei Zhang[1]

[1] *Cancer Genomics Laboratory, University of Texas M. D. Anderson Cancer Center, 1515 Holcombe Blvd., Unit 85, Houston, TX 77030, USA*
[2] *Sun Microsystems Laboratories, Palo Alto, CA, USA*
[3] *Department of Electrical Engineering, Texas A&M University, College Station, TX 77843, USA*
[4] *Departamento de Ciencia de Computacao, Universidade de Sao Paulo, Sao Paulo, Brazil*

**Correspondence to:*
*Ilya Shmulevich, Cancer Genomics Laboratory, University of Texas, M.D. Anderson Cancer Center, 1515 Holcombe Blvd, Unit 85, Houston, TX 77030, USA.*
*E-mail: is@ieee.org*

Received: 26 March 2003
Revised: 22 September 2003
Accepted: 3 October 2003

### Abstract

**Probabilistic Boolean networks (PBNs) have recently been introduced as a promising class of models of genetic regulatory networks. The dynamic behaviour of PBNs can be analysed in the context of Markov chains. A key goal is the determination of the steady-state (long-run) behaviour of a PBN by analysing the corresponding Markov chain. This allows one to compute the long-term influence of a gene on another gene or determine the long-term joint probabilistic behaviour of a few selected genes. Because matrix-based methods quickly become prohibitive for large sizes of networks, we propose the use of Monte Carlo methods. However, the rate of convergence to the stationary distribution becomes a central issue. We discuss several approaches for determining the number of iterations necessary to achieve convergence of the Markov chain corresponding to a PBN. Using a recently introduced method based on the theory of two-state Markov chains, we illustrate the approach on a sub-network designed from human glioma gene expression data and determine the joint steady-state probabilities for several groups of genes. Copyright © 2003 John Wiley & Sons, Ltd.**

**Keywords:** **genetic network; probabilistic Boolean network; steady-state analysis**

### Introduction

Modelling of genetic regulatory networks is becoming increasingly widespread for gaining insight into the underlying processes of living systems. The computational biology literature abounds in various modelling approaches, all of which have particular goals along with their strengths and weaknesses (de Jong, 2002; Hasty *et al.*, 2001; Smolen *et al.*, 2000). One important characteristic that is shared by some models is the ability to capture dynamic behaviour of the quantities that are being modelled. Some examples include Boolean networks (Kauffman, 1993; Huang, 1999; Somogyi and Sniegoski, 1996), dynamic Bayesian networks (Murphy and Mian, 1999; Smith *et al.*, 2002) and the recently-introduced probabilistic Boolean networks (PBNs) (Shmulevich *et al.*, 2002a, 2002b, 2002c, 2002d; Dougherty and Shmulevich, 2003; Zhou *et al.*, 2003; Kim *et al.*, 2002; Datta *et al.*, 2003).

A key aspect of the analysis of such dynamic systems is the determination of their steady-state (long-run) behaviour. Indeed, much of the power of prediction stems from this very knowledge. This paradigm closely parallels the mission of cancer professionals who aim to understand the ebb and flow of molecular events during cancer progression and to predict how disease will develop and how patients will respond to certain therapies. A specific example will illustrate this point. Comparing the gene expression profiles of human

Copyright © 2003 John Wiley & Sons, Ltd.

brain tumours of high grade (glioblastoma), mid-grade (anaplastic astrocytoma, anaplastic oligodendroglioma) and low grade (oligodendroglioma), a key gene expression event — elevated expression of insulin-like growth factor binding protein 2 (IGFBP2) occurring only in high-grade brain tumours — was identified (Fuller *et al.*, 1999). It can be envisioned that some gene expression events have been initiated at different stages at the low- and mid-grade brain tumours and may eventually have led to the (steady) state when IGFBP2 is activated. The faster these kinetics are, the faster is the progression of cancer. The goal is that the use of gene expression profiles, along with the construction of the gene networks using models such as PBNs, will make it possible to predict whether (and when) the convergence to events such as IGFBP2 activation state will occur.

By the same token, we also envision the ability to 'see' the next gene expression event when IGFBP2 is activated. Such an endeavour can be facilitated by laboratory experiments and prediction must be validated by existing knowledge of the biological system under investigation. For example, suppose IGFBP2 is activated by experimental manipulation. In order to determine the steady state behaviour of some other gene after IGFBP2 activation, biologists can select a relatively stable cell system that has a baseline level of IGFBP2. Then, IGFBP2 can be transfected into the cells and continuously expressed in the new or 'perturbed' cell system whose steady-state gene expression levels are of interest. Both cell systems can be surveyed in a genomic laboratory for the gene expression profiles. A comparison will shed light on how and where in the state space the network shifts, forming a basis for prediction. In one of the described comparisons carried out in our laboratory, cell invasion genes were activated in IGFBP2 overexpressed cells. This gene expression event is linked to a cellular attractor state — cell invasion (Wang, 2003).

In this paper, we concentrate on obtaining the steady-state (equilibrium) distribution of a PBN. This is a crucial task in many contexts. For instance, as was shown by Shmulevich *et al.* (2002b), the steady-state distribution is necessary in order to compute the so-called (long-term) *influence*, which is a measure of the impact of a gene on other genes. More 'influential' genes may possess a greater potential to regulate the dynamics of the network, as their perturbation can lead to significant 'downstream' effects. Shmulevich *et al.* (2002d) developed a methodology for altering the steady-state probabilities of certain states or sets of states with minimal modifications to the rule-based structure of the network. Reliable computation of steady-state distributions is crucial in this context as well. As another example, suppose we are interested in the long-term joint behaviour of several selected genes, i.e. we would like to obtain their limiting joint distribution. This information can supply answers to questions of the type: 'What is the probability that gene A will be expressed in the long run?' or 'What is the probability that gene B and gene C will *both* be expressed in the long run?' Steady-state analysis is necessary for answering such questions.

In this paper, we advocate the use of Monte Carlo simulation methods for making reliable inferences from steady-state analysis. A significant emphasis will be placed on the convergence rate of the Markov chain corresponding to the PBN, as it is crucial to ensure that the chain reaches stationarity before collecting information of interest. We also illustrate our methods by analysing sub-networks generated from human glioma gene expression data (the data set is described in Fuller *et al.*, 1999). The necessary background material, including definitions and some results related to PBNs, are available at **http://www3.interscience.wiley.com/cgi-bin/jabout/77002016/suppmat/index.html**.

### Steady-state analysis

Most approaches to steady-state analysis use the state transition matrix in some form or another. For the case of PBNs, this would consist of constructing the state-transition matrix $A$ given in Equation (4) in the Supplementary Material, and then applying numerical methods. A variety of approaches using iterative, projection, decompositional and other methods could potentially be used (Stewart, 1994). Unfortunately, however, in the case of PBNs, the size of the state space grows exponentially in the number of genes and becomes prohibitive for matrix-based numerical analysis of large networks. Even matrix-geometric methods, which have been successfully used for similar problems in non-linear signal processing (Shmulevich *et al.*, 1999), are

Copyright © 2003 John Wiley & Sons, Ltd. 

unsuitable due to the possibly irregular structure of the transition matrix.

On a more positive note, it should be recognized that even larger state spaces are commonly encountered in Markov chain Monte Carlo (MCMC) methods for many applications, including Markov random field modelling in image processing (Winkler, 1995), where efficient simulation and estimation are routinely performed. Thus, Monte Carlo methods represent a viable alternative to numerical matrix-based methods for obtaining steady-state distributions. Informally speaking, this consists of running the Markov chain for a sufficiently long time, until convergence to the stationary distribution is reached, and observing the proportion of time the process spends in the parts of the state space that represent the information of interest, such as the joint stationary distribution of several specific genes. A key factor is convergence, which to a large extent depends on the perturbation probability, $p$. In general, a larger $p$ results in quicker convergence, but making $p$ too large is not biologically meaningful.

In order for us to perform long-term analysis of the Markov chain corresponding to a PBN using Monte Carlo methods, we need to be able to estimate the convergence rate of the process. Only after we are sufficiently sure that the chain has reached its stationary distribution can we begin to collect information of interest. Typical approaches for assessing convergence are based on the second-largest eigenvalue of the transition probability matrix $A$. Unfortunately, as mentioned above, for even a moderate number of genes, obtaining the eigenvalues of the transition matrix may be impractical. Thus, it is advantageous to be able to determine the number of iterations necessary until satisfactory convergence is reached.

One approach for obtaining *a priori* bounds on the number of iterations is based on the so-called *minorization condition* for Markov chains (Rosenthal, 1995). The Supplementary Material, which is available at **http://www3.interscience.wiley.com/cgi-bin/jabout/77002016/suppmat/index.html**, discusses the minorization condition in the context of PBNs. Unfortunately, as we show in the Supplementary Material, the usefulness of such a result is rather limited. Even for a moderately small number of genes, the number of iterations, $k$, predicted by the bound is much too large to be useful in practice. We have also found (see Supplementary Material) that making any assumptions about the relative magnitudes of the probabilities of perturbation and transition via the selected Boolean functions is not likely to significantly improve the bound on convergence, and that it is only by having some information about the structure of the PBN itself that the bound can potentially be lowered. Thus, with limited ability to obtain *a priori* bounds, we now turn to diagnosing convergence to the steady-state distribution.

### Diagnosing convergence

In a practical situation, it is important to be able to empirically determine when to stop the chain and produce our estimates. For this purpose, there are a number of monitoring methods available (Cowles and Carlin, 1996; Robert, 1995). Consider, for example, the Kolmogorov–Smirnov test, a nonparametric test of stationarity that can be used to assess convergence.

### Kolmogorov–Smirnov test

When the chain is stationary, distributions $\pi^{(k_1)}$ and $\pi^{(k_2)}$ are the same for arbitrary times $k_1$ and $k_2$. Thus, given a sample $x^{(1)}, \ldots, x^{(T)}$, we can compare the two halves: $x^{(1)}, \ldots, x^{(T/2)}$ and $x^{(T/2+1)}, \ldots, x^{(T)}$. In order to correct for non-i.i.d. (correlated) samples, we introduce a 'batch size' $G$, leading to the construction of two (quasi-) independent samples (Robert and Casella, 1999). We thus select subsamples $x_1^{(G)}, x_1^{(2G)}, \ldots$ and $x_2^{(G)}, x_2^{(2G)}, \ldots$ and use the Kolmogorov–Smirnov statistic with the lexicographical ordering to define the indicator:

$$K = \frac{1}{M} \max_{\eta} \left| \sum_{g=1}^{M} 1_{[0\cdots 0,\eta)} \left( x_1^{(gG)} \right) - \sum_{g=1}^{M} 1_{[0\cdots 0,\eta)} \left( x_2^{(gG)} \right) \right|, \quad (1)$$

where the maximum is over the state space, the vertices of an $n$ dimensional Boolean hypercube. As $\sqrt{M}K$ has the cumulative distribution function $R(x) = 1 - \sum_{k=1}^{\infty} (-1)^{k-1} e^{-2k^2x^2}$ (Robert and Casella, 1999), the corresponding $p$ value can be computed for each $T$ until it reaches a desired level.

Copyright © 2003 John Wiley & Sons, Ltd. 

This can also be used to assess convergence for a selected group of genes $j_1, \cdots, j_m$ by replacing state vectors $x_1^{(gG)}$ and $x_2^{(gG)}$ in (1) with just the vectors of their $j_1$th, $\cdots, j_m$th coordinates and modifying the domain of $\eta$ into the hypercube over these coordinates only. For example, if only the distribution for the first gene is of interest, i.e. $\pi(x(1) = 0)$, the maximum in (1) degenerates into just an absolute difference of the numbers of zeros in the first coordinate between the two samples.

### Two-state Markov chain approach

We also employ a promising approach proposed by Raftery and Lewis (1992). This method reduces the study of the convergence of the chain to the study of the convergence of a two-state Markov chain. Suppose that we are interested in knowing the steady-state probability of the event (gene A is ON and gene B is OFF). Then, we can partition the state space into two disjoint subsets such that one subset contains all states on which the event occurs and the other subset contains the rest of the states. Consider the two 'meta-states' corresponding to these two subsets. Although the sequence of these meta-states does not form a Markov chain in itself, it can be approximated by a first-order Markov chain if every $k$ state from the original Markov chain is discarded (i.e. the chain is subsampled). It turns out in practice that $k$ is usually equal to 1, meaning that nothing is discarded and the sequence of meta-states is treated as a homogeneous Markov chain (see Raftery and Lewis for details) with transition probabilities $\alpha$ and $\beta$ between the two meta-states. Using standard results for two-state Markov chains, it can be shown that the 'burn-in' period (the number of iterations necessary to achieve stationarity), $m_0$, satisfies:

$$m_0 \geq \log\left(\frac{\varepsilon(\alpha+\beta)}{\max(\alpha,\beta)}\right) \Big/ \log(1-\alpha-\beta) \qquad (2)$$

We set $\varepsilon = 0.001$ in our experiments. In addition, it can be shown that the minimum total number of iterations, $N$, necessary to achieve a desired accuracy, $r$ (we use $r = 0.01$ in our experiments), is:

$$N = \frac{\alpha\beta(2-\alpha-\beta)}{(\alpha+\beta)^3}\left(\frac{r}{\Phi\left(\frac{1}{2}(1+s)\right)}\right)^{-2} \qquad (3)$$

where $\Phi(\cdot)$ is the standard normal cumulative distribution function and $s$ is a parameter that we set to 0.95 in our experiments. For detailed explanations of the 'precision' parameters $\varepsilon$, $r$, and $s$, see Raftery and Lewis (1992). The question becomes how to estimate the transition probabilities $\alpha$ and $\beta$, as these are unknown. The solution is to perform a test run from which $\alpha$ and $\beta$ can be estimated and from which $m_0$ and $N$ can be computed. Then, another run with the computed burn-in period $m_0$ and the total number of iterations $N$ is performed and the parameters $\alpha$ and $\beta$ are re-estimated, from which $m_0$ and $N$ are recomputed. This can be done several times in an iterative manner until the estimates of $m_0$ and $N$ are smaller than the number of iterations already achieved. We have used this method to determine the steady-state probabilities of some genes of interest from our gene expression data set, as described below.

### Experimental results

Using a human glioma gene expression data set, as described in Fuller *et al.* (1999), we constructed a small sub-network consisting of 15 genes, shown in Figure 1. The entire 597-gene network was inferred using the coefficient of determination (Dougherty *et al.*, 2000), as described in (Shmulevich *et al.*, 2002a). The algorithm for building a sub-network starting from so-called 'seed' genes, which uses influences of genes (Shmulevich *et al.*, 2002a) and ensures that the sub-network functions fairly autonomously from the rest of the genes, is described by Hashimoto *et al.* (A direct-graph algorithm to grow genetic regulatory subnetworks from seed genes based on strength of connection: Submitted for publication). The numbers next to the arrows in Figure 1 indicate the influences.

We analysed the joint steady-state probabilities of several combinations of two genes: Tie-2 and NF$\kappa$B; Tie-2 and TGF$\beta_3$; and TGF$\beta_3$ and NF$\kappa$B. For example, for Tie-2 and NF$\kappa$B, the two-state Markov chain method described above, when applied to an initial run of 10 000 iterations, produced a burn-in period of $m_0 = 87$ and a total number of iterations of $N = 48\,268$. The transition probabilities $\alpha$ and $\beta$ were both approximately equal to 0.03. The perturbation probability $p$ was set to 0.001. When we ran the network for another 38 268 steps, the recomputed values of $m_0$ and $N$

Copyright © 2003 John Wiley & Sons, Ltd. 

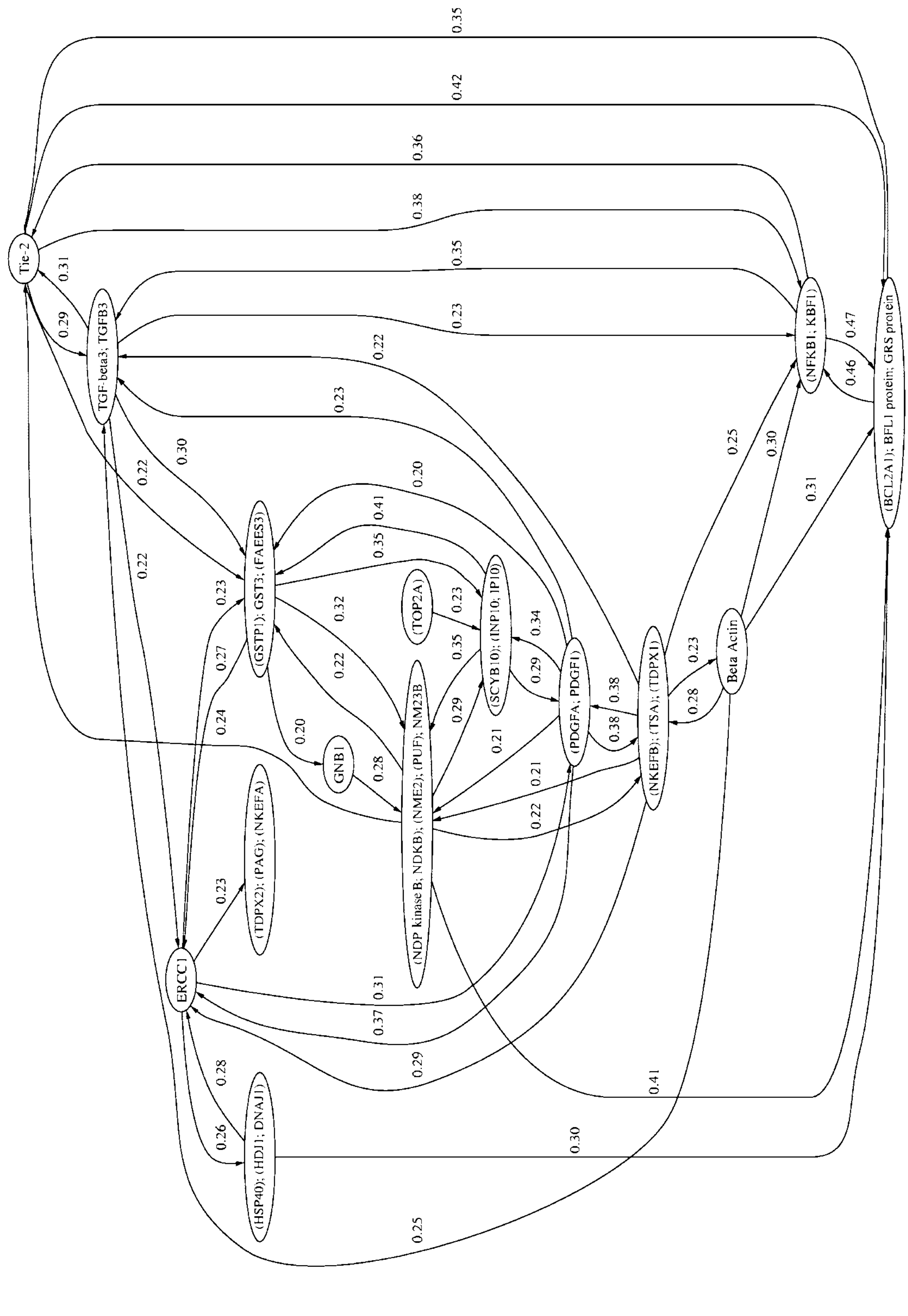

**Figure 1.** The sub-network that is used for the steady-state analysis. The numbers next to the arrows indicate the influences (Shmulevich *et al.*, 2002a). Only those influences are that greater than 0.2 are shown

Copyright © 2003 John Wiley & Sons, Ltd. 

**Table 1.** Steady-state analysis of several pairs of genes

| Tie-2 | *NFκB* | % | Tie-2 | *TGFβ3* | % | *TGFβ3* | *NFκB* | % |
|---|---|---|---|---|---|---|---|---|
| OFF | OFF | 15.68 | OFF | OFF | 14.75 | OFF | OFF | 10.25 |
| OFF | ON | 41.58 | OFF | ON | 42.50 | OFF | ON | 12.47 |
| ON | OFF | 9.21 | ON | OFF | 7.96 | ON | OFF | 14.64 |
| ON | ON | 31.53 | ON | ON | 32.78 | ON | ON | 60.65 |

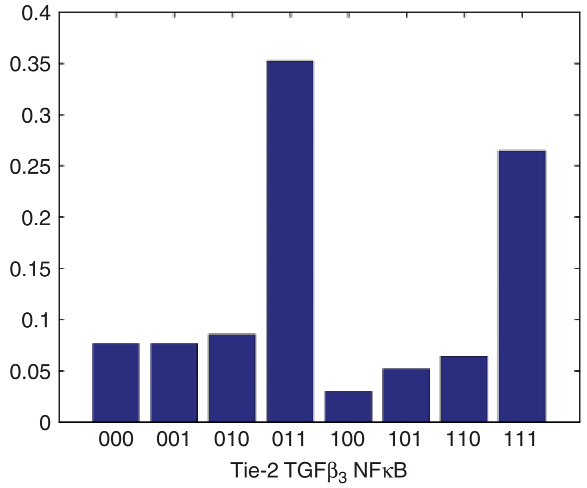

**Figure 2.** Steady-state analysis of all three genes: Tie-2, TGF$\beta_3$ and NF$\kappa$B. The gene combinations are coded in binary, e.g. 010 means that Tie-2 is OFF, TGF$\beta_3$ is ON and NF$\kappa$B is OFF. The bars show the joint steady-state probabilities were 91 and 50 782, respectively. Running the network for another 3000 iterations was sufficient for the given accuracy and the steady-state probabilities of these two genes could be determined. The steady-state probabilities for all pairs of considered genes are shown in Table 1 as percentages. Figure 2 shows the joint steady-state probabilities for all three of these genes using a bar graph.

Tie-2 is a receptor tyrosine kinase expressed on the endothelial cells. Its two ligands, angiopoietin 1 and 2, bind Tie-2 and regulate vasculogenesis (Sato *et al.*, 1993), an important process in embryonic development and tumour development. Other related regulators for vasculogenesis are VEGF and VEGF receptors, which are often overexpressed in the advanced stage of gliomas (Cheng *et al.*, 1996). Although no experimental evidence supports a direct transcriptional regulation of those regulators by the transcriptional factor NF$\kappa$B, which is also frequently activated in glioma progression (Hayashi *et al.*, 2001) as predicted in this analysis, the results show that NF$\kappa$B, at least indirectly, influences the expression of Tie-2 expression. Thus, it may not be surprising that when NF$\kappa$B is on, Tie-2 is on about $31.53/(41.58 + 31.53) = 43\%$ of the time. Because Tie-2 is only one of the regulators for the important vasculogenesis in glioma progression, it is consistent that our analysis of long-term (steady-state) gene expression activities shows that about 40% of the time Tie-2 is on. In contrast, NF$\kappa$B is on 73% of the time, implying that fewer redundancies exist for NF$\kappa$B activity.

Interestingly, a similar relationship exists between Tie-2 and TGF$\beta_3$, as can be seen by comparing the percentages in columns 3 and 6 of Table 1. This suggests that TGF$\beta_3$ and NF$\kappa$B are more directly linked, which is also shown in the last three columns of the table (60% of the time, they are both on). This relationship is very likely because TGF$\beta_1$, a homologue of TGF$\beta_3$, was shown to have a direct regulatory relationship with NF$\kappa$B (Arsura *et al.*, 1996).

### Concluding remarks

We have focused on the important problem of steady-state analysis of probabilistic Boolean networks. For even $n = 20$ genes, working with $2^{20} \times 2^{20}$ matrices becomes cumbersome and quickly prohibitive for larger $n$. However, Monte Carlo techniques can be successfully used as long as we are sufficiently confident that the Markov chain corresponding to the PBN has converged to its equilibrium distribution. Moreover, Monte Carlo techniques exhibit favorable scaling behaviour with respect to the number of genes. Despite the fact that the size of the state space grows exponentially with $n$, efficient steady-state analysis can still be carried out. We again note that MCMC methods on images containing $512 \times 512 = 2.6214 \times 10^5$ pixels, resulting in state spaces on the order of $10^{78,912}$

Copyright © 2003 John Wiley & Sons, Ltd. 

for binary models, can nonetheless be effectively performed. The actual inference step, however, is more challenging. When selecting the best predictor genes (inputs) for one target gene (output), using some measure such as the coefficient of determination, we face a combinatorial explosion: for 600 genes, there are $\binom{600}{3} = 3.582 \times 10^7$ possible three-gene predictors to be tested for each of the 600 genes. So far, we have relied on massively-parallel supercomputers for carrying out the inference. However, other sub-optimal efficient methods are now becoming available (Hashimoto *et al.*, 2003).

Using a recently developed method for determining the number of iterations necessary for satisfactory convergence, we have performed steady-state analysis of several genes of interest in a small PBN sub-network generated from real gene expression data. The results seem to be in consonance with what is known about these genes, especially as it relates to tumourigenesis. Clearly, such a modelling approach may prove to be very useful for generating many valuable hypotheses for biologists to test in the laboratories, which is one of the goals of modelling in computational biology.

### References

Arsura M, Wu M, Sonenshein GE. 1996. TGF$\beta$1 inhibits NF-$\kappa$B/Rel activity inducing apoptosis of B cells: transcriptional activation of I$\kappa\beta\alpha$. *Immunity* **5**(1): 31–40.

Cheng SY, Huang HJ, Nagane M *et al.* 1996. Suppression of glioblastoma angiogenicity and tumorigenicity by inhibition of endogenous expression of vascular endothelial growth factor. *Proc Natl Acad Sci USA* **93**(16): 8502–8507.

Cowles MK, Carlin BP. 1996. Markov chain Monte Carlo convergence diagnostics: a comparative study. *J Am Statist Assoc* **91**: 883–904.

Datta A, Choudhary A, Bittner M, Dougherty ER. 2003. External control in Markovian genetic regulatory networks. *Machine Learning* **52**: 169–181.

de Jong H. 2002. Modeling and simulation of genetic regulatory systems: a literature review. *J Comput Biol* **9**(1): 67–103.

Dougherty ER, Shmulevich I. 2003. Mappings between probabilistic Boolean networks. *Signal Process* **83**(4): 799–809.

Dougherty ER, Kim S, Chen Y. 2000. Coefficient of determination in non-linear signal processing. *Signal Process* **80**(10): 2219–2235.

Fuller GN, Rhee CH, Hess KR, *et al.* 1999. Reactivation of insulin-like growth factor binding protein 2 expression in glioblastoma multiforme: a revelation by parallel gene expression profiling. *Cancer Res* **59**: 4228–4232.

Goldberg D. 1989. *Genetic Algorithms in Search, Optimization, and Machine Learning*. Addison-Wesley: Reading, MA.

Hashimoto RF, Dougherty E, Brun M *et al.* 2003. Efficient selection of feature sets possessing high coefficients of determination based on incremental determinations. *Signal Process* **83**(4): 695–712.

Hasty J, McMillen D, Isaacs F, Collins JJ. 2001. Computational studies of gene regulatory networks: *in numero* molecular biology. *Nature Rev Genet* **2**(4): 268–279.

Hayashi S, Yamamoto M, Ueno Y, *et al.* 2001. Expression of nuclear factor-$\kappa$B, tumor necrosis factor receptor type 1, and c-Myc in human astrocytomas. *Neurol Med Chir (Tokyo)* **41**(4): 187–195.

Huang S. 1999. Gene expression profiling, genetic networks, and cellular states: an integrating concept for tumorigenesis and drug discovery. *J Mol Med* **77**(6): 469–480.

Kauffman SA. 1993. *The Origins of Order: Self-organization and Selection in Evolution*. Oxford University Press: Oxford.

Kim S, Dougherty ER, Chen Y, *et al.* 2000a. Multivariate measurement of gene expression relationships. *Genomics* **67**: 201–209.

Kim S, Dougherty ER, Bittner ML *et al.* 2000b. General non-linear framework for the analysis of gene interaction via multivariate expression arrays. *J Biomed Optics* **5**(4): 411–424.

Kim S, Dougherty ER, Cao N *et al.* 2002. Can Markov chain models mimic biological regulation? *J Biol Syst* **10**(4): 431–445.

Murphy K, Mian S. 1999. *Modelling Gene Expression Data using Dynamic Bayesian Networks*. Technical Report, University of California: Berkeley.

Raftery AE, Lewis S. 1992. How many iterations in the Gibbs sampler? In *Bayesian Statistics,* vol 4, Berger JO *et al.* (eds). Oxford University Press: Oxford; 763–773.

Robert CP. 1995. Convergence control techniques for Markov chain Monte Carlo algorithms. *Statist Sci* **10**(3): 231–253.

Robert CP, Casella G. 1999. *Monte Carlo Statistical Methods*. Springer: New York.

Rosenthal JS. 1995. Minorization conditions and convergence rates for Markov chain Monte Carlo. *J Am Statist Assoc* **90**(430): 558–566.

Sato TN, Qin Y, Kozak CA, Audus KL. 1993. Tie-1 and tie-2 define another class of putative receptor tyrosine kinase genes expressed in early embryonic vascular system. *Proc Natl Acad Sci USA* **90**(20): 9355–9358.

Shmulevich I, Yli-Harja O, Egiazarian K, Astola J. 1999. Output distributions of recursive stack filters. *IEEE Sign Process Lett* **6**(7): 175–178.

Shmulevich I, Dougherty ER, Kim S, Zhang W. 2002a. Probabilistic Boolean networks: a rule-based uncertainty model for gene regulatory networks. *Bioinformatics* **18**(2): 261–274.

Shmulevich I, Dougherty ER, Zhang W. 2002b. Gene perturbation and intervention in probabilistic Boolean networks. *Bioinformatics* **18**(10): 1319–1331.

Shmulevich I, Dougherty ER, Zhang W. 2002c. From Boolean to probabilistic Boolean networks as models of genetic regulatory networks. *Proc IEEE* **90**(11): 1778–1792.

Shmulevich I, Dougherty ER, Zhang W. 2002d. Control of stationary behavior in probabilistic Boolean networks by means of structural intervention. *J Biol Syst* **10**(4): 431–445.

Smith VA, Jarvis ED, Hartemink AJ. 2002. Evaluating functional network inference using simulations of complex biological systems. *Bioinformatics* **18**: s216–s224.

Smolen P, Baxter DA, Byrne JH. 2000. Mathematical modeling of gene networks. *Neuron* **26**: 567–580.

Copyright © 2003 John Wiley & Sons, Ltd. 

Somogyi R, Sniegoski C. 1996. Modeling the complexity of gene networks: understanding multigenic and pleiotropic regulation. *Complexity* **1**: 45–63.

Stewart WJ. 1994. *Introduction to the Numerical Solution of Markov Chains*. Princeton University Press: Princeton.

Wang H, Wang H, Shen W *et al*. 2003. Insulin-like growth factor binding protein 2 enhances glioblastoma invasion via activation of invasion enhancing genes. *Cancer Res* **63**: 4315–4321.

Winkler G. 1995. *Image Analysis, Random Fields and Dynamic Monte Carlo Methods: A Mathematical Introduction*. Springer Verlag: Berlin.

Zhou X, Wang X, Dougherty ER. 2003. Construction of genomic networks using mutual-information clustering and reversible-jump Markov-chain-Monte-Carlo predictor design. *Sign Process* **80**(10): 745–761.

Copyright © 2003 John Wiley & Sons, Ltd. 
